# Major drivers of health inequalities in later life and future research needs

**DOI:** 10.1186/1753-6561-7-S4-S5

**Published:** 2013-08-23

**Authors:** Carol Jagger

**Affiliations:** 1 Institute for Ageing and Health, Newcastle University, Newcastle u. T., UK

## 

Europe is the oldest region in the world and its life expectancy is currently increasing at around about 12 months every 5 years. When added to population re-structuring as a result of demographic change, this means that by 2060 30% of the European population will be aged 65 years and over and with those aged 80 and over being the fastest growing age group. The European Union is placing a high priority on healthy ageing and its key indicator is Healthy Life Years (HLY). It is already obvious from the variation in HLY that not all European populations are ageing healthily. For example in France, life expectancy at age 50 for women is 22.9 years, with 9.9 years of healthy life. In comparison women’s life expectancy at age 50 in Latvia is 17.2 years, with only 4.3 years of healthy life.

In 2009 the European Commission funded the FUTURAGE project, whose aim was to develop a definitive road map for ageing research for Europe for the next 10-15 years. Seven major research priority themes were identified by FUTURAGE according to a standard format covering: the significance of the theme; the fundamental insights necessary for future research; an overview of current research knowledge; and the main priority topics within the general theme (full report available at http://futurage.group.shef.ac.uk/road-map-launch-conference.html). Here we focus on one of these seven themes, that of ‘Unequal Ageing’.

Six challenges related to future European ageing research were identified in the area of unequal ageing: Monitoring inequalities; Health in work and retirement; Inequalities and discrimination on health; Ageing and migration; Focus on the very old; and Inequalities and discrimination in the labour market. These challenges, as well as examples of specific research questions, can be described in the context of a life course perspective and the World Health Organisation model of health transitions.

FUTURAGE represented the most extensive consultation ever conducted in this research area, and it included all major stakeholders and active users of ageing research. ‘Unequal Ageing’ was identified as one of the main research priorities for the road map and within this the challenge to monitor inequalities across Europe. To be able to address this challenge demands not only a concerted collection of truly harmonised measures across Europe, but also that they are appropriate at a country or regional level.

